# Environmental impact of food consumption and sociodemographic factors in Northern Norway through an intersectional lens: a cross-sectional study

**DOI:** 10.1186/s12889-025-23899-3

**Published:** 2025-08-30

**Authors:** Bahar Kucuk, Charlotta Rylander, Monica Hauger Carlsen, Marie Wasmuth Lundblad, Lene Frost Andersen, Guri Skeie

**Affiliations:** 1 Department of Community Medicine, Faculty of Health Sciences, UiT The Arctic University of Norway, Hansine Hansens Veg 18, Tromsø, 9019 Norway; 2 Department of Nutrition, Institute of Basic Medical Sciences, Faculty of Medicine, University of Oslo, Oslo, 0316 Norway

**Keywords:** Environmental impact, Sustainable diet, Food consumption, Intersectionality, Norway

## Abstract

**Background:**

Food systems contribute significantly to environmental degradation. The interplay of sociodemographic factors influences food choices and thus, the environmental impacts of diet. This study investigated the environmental impact of food consumption in Northern Norway, focusing on intersectional dynamics.

**Methods:**

A cross-sectional design was employed using data from the Tromsø Study. The diet was assessed using a food frequency questionnaire, and the environmental impacts of the diet were estimated for greenhouse gas emissions, water use, land use, acidification, and eutrophication using a Norwegian life cycle analysis food database. Multiple linear regression analyses were performed to examine the associations with sociodemographic variables using three-way interactions with sex, education, and income in an inter-categorical intersectionality framework. Pairwise contrasts were calculated to assess the mean differences between interacting groups.

**Results:**

The typical diet in Northern Norway substantially impacts the environment, with dairy products being the primary contributor. When controlling for energy intake, age was inversely linked to the environmental impact, whereas a higher body mass index corresponded to a greater environmental impact. No clear association with sex was observed, and the associations among environmental impact, education, and income varied. Including intersectional analyses did not significantly improve the explanatory power of the models. Although a few pairwise comparisons were statistically significant, the effect sizes were generally small.

**Conclusion:**

The study underscores the complex dynamics of dietary habits and sociodemographic factors in shaping the environmental impact of food consumption. The findings are important to develop approaches that balance sustainability perspectives and the diverse needs of the population in Northern Norway.

**Graphical Abstract:**

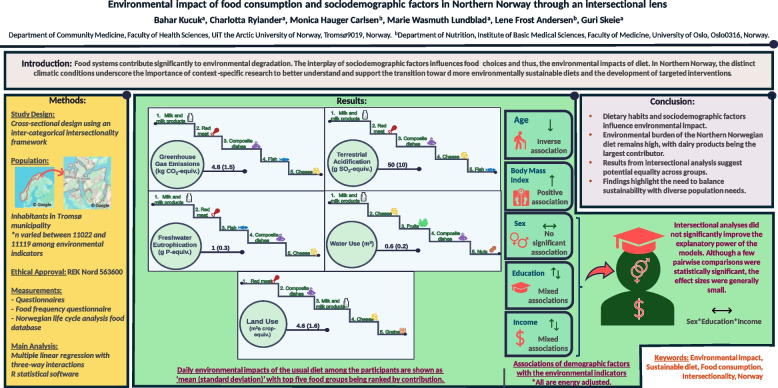

**Supplementary Information:**

The online version contains supplementary material available at 10.1186/s12889-025-23899-3.

## Background

The degradation of environmental and natural resources at an accelerated pace is a major global challenge, and food systems play a significant role [1]. Agricultural production accounts for 40% of global land use and 70% of freshwater withdrawals, whereas global food systems account for approximately one-third of global greenhouse gas emissions (GHGE) [2–4]. The increasing growth of the human population enlarges the demand for food, and the predicted expansion of the world’s population makes our current food systems unsustainable as the status quo already pushes the earth well beyond most of its planetary boundaries [1, 5]. The global food system contributes to and is impacted by environmental changes, as well as being one of the key solutions to tackle these challenges [5].

Food consumption patterns vary globally, displaying regional, inter-, and intra-country variations [6–8]. As food production is largely driven by food consumption and dietary habits, a profound transformation of the food system is not possible by considering the production side alone, and efforts are needed from both the supply and demand sides across all aspects of the global food system, from the field/fjord to the plate [9–11]. Individual food choices are driven by many factors other than concerns about health and the environment, such as accessibility, acceptability, affordability, and demographic and social characteristics [1, 3, 5, 9, 12–14].

Further, it is essential to consider the interplay of these multiple overlapping social determinants, rather than examining them in isolation, as these factors jointly shape the dietary choices [15, 16]. The interactions between determinants occur within interconnected systems and power structures, resulting in inequalities [17–20]. Hence, (dis)advantages from multiple social dimensions are not additive; instead, they intersect and amplify one another, leading to larger effects than the simple combination [21, 22]. Both The World Health Organization (WHO) and the EatLancet Commission underscore the need to address the major gaps between health, sustainability, and justice [1, 2]. Therefore, it is crucial to examine the relationship between social differences and dietary sustainability, as well as to test whether multiplicative differences exist [1, 2, 23]. To transition towards more sustainable healthy diets, a clear understanding of the current dietary environmental impacts in relation to sociodemographic factors and their interplay is warranted [3, 9, 24, 25].

Studies have assessed the environmental sustainability of individual foods and overall diets; however, it is mostly limited to GHGE [5, 6, 26, 27]. While dietary contributions to the environment regarding GHGE, eutrophication, and acidification vary strongly among food groups [13, 28], evidence from previous studies also occasionally demonstrates conflicting associations between dietary patterns and environmental outcomes. For example, adherence to healthy diets or indexes such as Dietary Approaches to Stop Hypertension (DASH), Mediterranean diet, or Healthy Eating Index (HEI) has shown varied results: some studies report inverse associations with GHGE [29–35], while others find positive associations [32, 36] or observed no linear associations [31]. For land use, some studies show an inverse relationship [33, 36], while others showed no clear pattern [31]. Water use also varies, with some studies showing higher use with adherence to Mediterranean diet [33–35], while others find no linear association [31] reflecting the complexity of dietary sustainability.

As stated in the latest Nordic Nutrition Recommendations report, the environmental impacts of current diets in Nordic countries mostly surpass the levels necessary to remain within most planetary boundaries despite commitments to international environmental agreements [13, 37]. As highlighted in a recent study by Lengle et al. [38] evaluating the dietary environmental impact among Norwegian adults, given the exceeded targets and the Nordic region's strong commitment to global sustainability efforts, it is crucial to assess the sustainability of current diets to identify more effective mitigation strategies and pathways for reducing environmental impacts. However, no studies have examined the association between food consumption in Northern Norway and environmental health.

Northern Norway has a rich fish industry, grass-based animal husbandry, and close access to berries and locally grown root vegetables [39]. However, climatic conditions limit the growing season, choice of crops, and the production of cereals, fruits, and vegetables with self-sufficiency rate being approximately 7.4% in Troms County [13, 37, 39, 40]. Often long distances exist between producers and consumers; therefore, food selection in local stores can be limited or unaffordable [41]. WHO, Food and Agriculture Organization of the United Nations (FAO), and the Norway Climate Action Plan 2021–2030 highlight that context-specific research on the environmental impact of diets in different demographic groups is essential for the transition to more environmentally sustainable diets and the creation of targeted interventions [5, 9, 13, 28, 37, 41–43].

Therefore, this study aimed to i) calculate the environmental impact of food consumption in Northern Norway in terms of GHGE, terrestrial acidification, freshwater eutrophication, water use, and land use, and ii) investigate the associations between these environmental impacts and sociodemographic factors using an intersectional lens.

## Materials and methods

### Theoretical framework

**Fig. 1:**
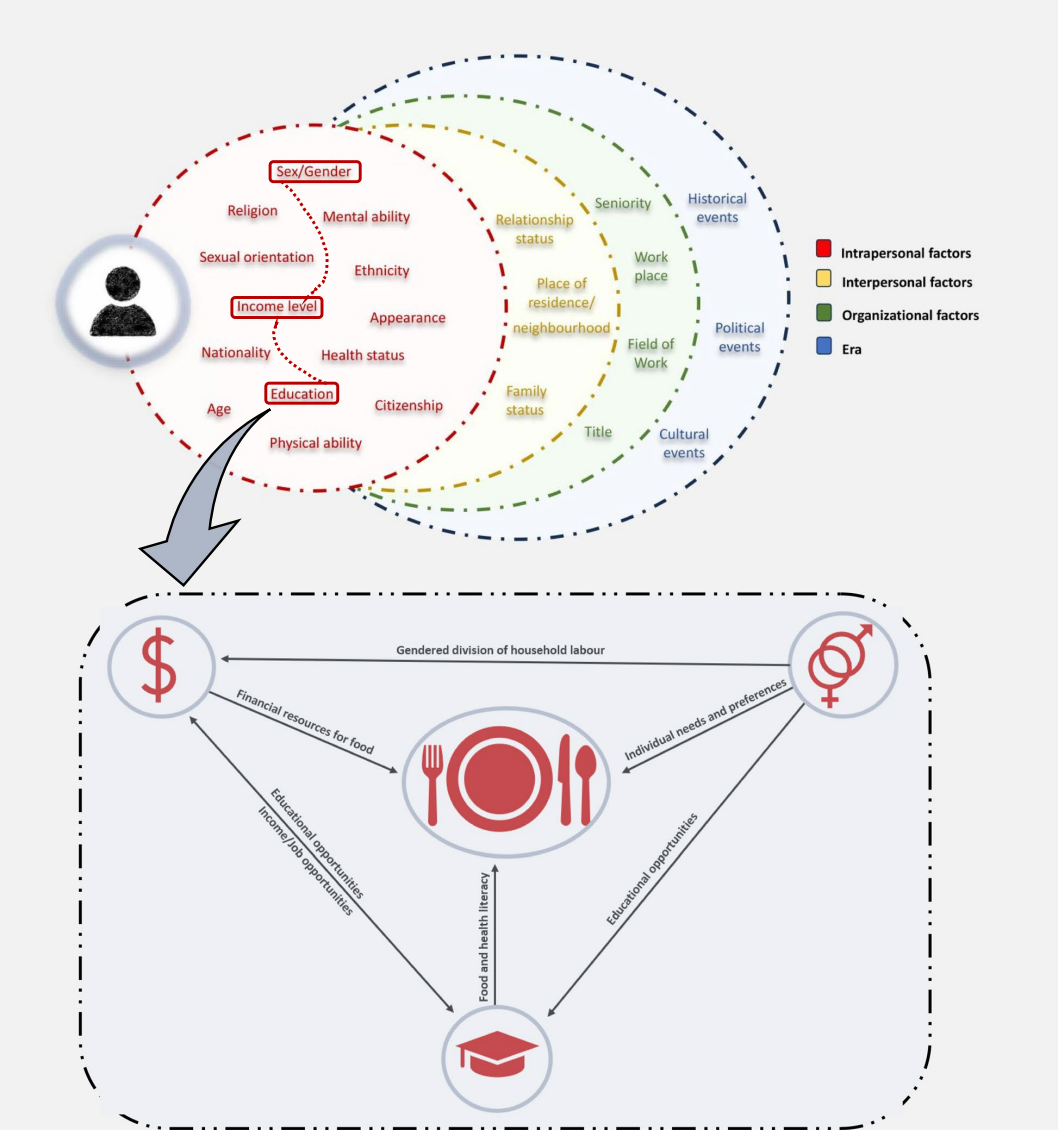
Intersectionality Framework. The figure illustrates the intersectional framework employed in our study. The first section depicts how intrapersonal, interpersonal, organizational, and broader societal factors interact both within and across levels, emphasizing the interconnected and dynamic nature of identity and experience. The zoomed-in section highlights some of the specific pathways between the pre-selected categories for our intersectional analyses (sex, education, and income), visualizing the complex interplay of these factors in shaping food-related behaviors and, consequently, dietary sustainability [41, 46–48]. The figure was adapted from Scottish Government [20] and Thomas et al. [49]

An intersectional framework was adopted for this study with the study being technically intersectional in addition to using intersectional theorizing (Fig. 1). The framework aims to understand how distinct experiences are shaped through the interaction of multiple forms of disadvantage, privilege, and power, rather than summing different kinds of inequality or positioning different groups against each other to assess who is most marginalized or disadvantaged [20, 23, 44]. An inter-categorical intersectionality approach was specifically used in this study, allowing us to examine outcomes across different social positions [45]. Among the many determinants affecting food consumption, we focused on sex, education, and income because of their interconnected influence on dietary sustainability [41, 46–48]. By focusing on this three-way interaction, we aim to capture the multiplicative and compounding effects, offering a more nuanced understanding of how structural inequalities shape food consumption and dietary sustainability.

### Study design and population

This cross-sectional study adhered to the Strengthening the Reporting of Observational Studies in Epidemiology (STROBE) checklist for cross-sectional studies to ensure transparent reporting [50].

This study includes data from the seventh survey of the Tromsø Study (Tromsø7), an ongoing population-based cohort study in the municipality of Tromsø, Northern Norway [51]. The Tromsø Study was performed in accordance with the 1964 Helsinki declaration and its later amendments [51]. Tromsø7 was approved by the Regional Committee for Medical Research Ethics (REK North ref. 2014/940) and the Norwegian Data Protection Authority [51] as well as the current study (REK Nord 563600). Informed written consent was obtained from all participants [51].

The survey was conducted between 2015 and 2016 [51]. All inhabitants of Tromsø municipality aged ≥ 40 years were invited, and the attendance was 65% [51]. The study sample comprised those who answered a food frequency questionnaire (FFQ) (n = 15, 146). Those who completed less than 90% of the FFQ, based on the number of questions skipped, (n = 3,689) and those with missing data (n = 8) were excluded. The interquartile range (IQR) method was used to identify and remove outliers, and all data points that fell below the lower quartile minus 1.5 times the IQR or above the upper quartile plus 1.5 times the IQR were excluded across all impact categories [52, 53].

**Fig. 2:**
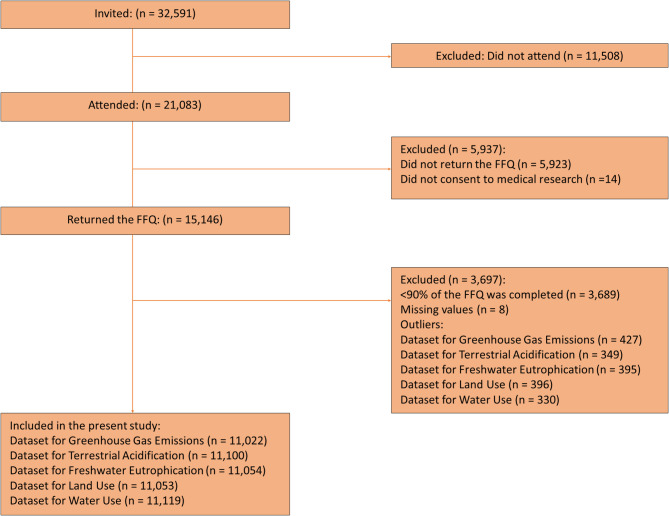
Participant flow chart

The final study sample is shown in a participant flowchart (Fig. 2).

### Measurements

Participants in Tromsø7 completed questionnaires regarding demographic and health characteristics and attended clinical examinations, which included anthropometric measurements [51]. Information on age, sex, education, income, body mass index (BMI), physical activity, and smoking status was used, with the latter two included only as adjustment variables. Age was categorized into five groups: 40–49, 50–59, 60–69, 70–79, and 80 + years. Sex was used as a binary variable, males and females. The highest level of education completed was assessed in four categories: primary/up to ten years, secondary (a minimum of three additional years), short tertiary (college/university less than four years), and long tertiary (college/university four years or more). Income was assessed based on the household’s total taxable income for the previous year, and it was categorized into five groups: very low (≤ 350,000 NOK), low (350,000–550,000 NOK), moderate (550,000–750,000 NOK), high (750,000–1,000,000 NOK), very high (1,000,000 NOK +). BMI was calculated as weight in kilograms (kg) divided by height in meters (m) squared and was categorized into three groups: normal weight (< 25.0 kg/m^2^), overweight (25.0–29.9 kg/m^2^) and obese (≥ 30.0 kg/m^2^). Few participants were underweight (< 18.5 kg/m^2^) (n = 56) and therefore merged with the normal-weight category. Physical activity was assessed in four categories with a validated scale [54]: sedentary, light, moderate, and vigorous. Smoking status was assessed in three categories: never, previous, and current.

All participants received a validated FFQ [55, 56] to complete at site or at home and to be returned by postal mail in a pre-paid envelope. The FFQ included questions about the frequency and amount of intake of 244 food items, dishes, and beverages. Dietary consumption during the previous year was collected retrospectively from FFQs, and total energy intake values (kJ/d) were calculated using the existing food composition and nutrient calculation system (KBS) at the University of Oslo (UiO) [57]. A full version of the FFQ is available on the Tromsø Study webpage [51].

The impact of diet was estimated for five environmental impact categories using a Norwegian life cycle analysis (LCA) food database comprising values for GHGE (kg CO_2_-equiv.), water use (m^3^), land use (m^2^a crop-equiv.), terrestrial acidification (g SO_2_-equiv.), and eutrophication values for freshwater (g P-equiv.) resources [58]. The Norwegian LCA food database was compiled from published LCA studies [58]. LCA assesses the environmental impact of a product on resource extraction, processing, packaging, distribution, usage, and end-of-life disposal [3, 6]. The Norwegian LCA food database is integrated into KBS, at the Department of Nutrition, UiO [57, 59]. Detailed information regarding the LCA food database has been given elsewhere [58].

### Data analysis

Twenty-two different food groups were identified from the FFQ. The categorization considered the variations in documented environmental impacts of the food items in the literature, alongside the regional consumption patterns, ensuring that the groups were defined in a manner that accurately reflecting both their environmental profiles and the dietary habits prevalent within the population. The environmental impact per person per day was calculated for each impact category, and the contribution of each food group to each environmental impact category was computed.

The means, standard deviations, and quartiles were calculated for each environmental impact category. Differences in participant characteristics were tested using Pearson’s chi-square test. Analysis of variance (ANOVA) tests were conducted to compare the mean differences in the environmental impact categories across the sociodemographic and BMI variables, and Tukey’s post-hoc comparisons were used when needed. Multiple linear regression analyses were performed using various indicators as dependent variables to examine the association between each environmental impact category and the variables of interest. The independent variables were age, sex, educational level, income, and BMI. Observations with missing values for any variable were excluded from the analysis. Energy intake was included as an adjusting factor in each model, as well as physical activity and smoking status, to account for possible confounding effects.

For intersectional analyses, models with three-way interactions were used in the multiple regression analysis, and the combined effects of the interactions were checked using a likelihood ratio test. In addition, the marginal means for the interactions were calculated for both sexes and the highest and lowest levels of education and income. Furthermore, contrasts were calculated using a pairwise function to determine the predicted differences between these pairs of interacting groups. To avoid the increased risk of chance findings due to multiple interaction groups, the relatively conservative Bonferroni correction was used for *p*-values.

An alpha level of 0.05 was selected for significance throughout the study. All statistical analyses were performed using R statistical software versions 4.2.2. and 4.4.0 [60].

## Results

### Characteristics of the study sample

This study sample comprised participants who completed ≥ 90% of the FFQ. Across age groups; participation was relatively evenly distributed, with slightly fewer participants in the oldest age group (80 +). A higher percentage of respondents were female, overweight, former smokers, and engaged in light physical activity. The highest educational and income levels were slightly predominant. No major differences were observed between participants who completed ≥ 90% of the FFQ and those who completed < 90%, except that the latter group was generally older and had lower education and income status (S1).

### Environmental impacts of the diets

The estimated daily environmental impacts of the average usual diet for each impact category are represented in Table 1.

**Table 1:** Daily environmental impact of the usual diet of participants in Tromsø7*: stratified by age, sex, education, and income

	***GHGE***	***TA***	***FE***	***LU***	***WU***
***Overall***	**4.8 (1.5)** **n = 11,022**	**50 (10)** **n = 11,100**	**1.0 (0.3)** **n = 11,054**	**4.6 (1.6)** **n = 11,053**	**0.6 (0.2)** **n = 11,119**
*Q1; Q3*	3.7; 5.8	30; 60	0.8; 1.3	3.5; 5.6	0.4; 0.7
*Mean EI*	9.5 MJ	9.5 MJ	9.5 MJ	9.5 MJ	9.6 MJ
*Age*					
*40–49*	5.0 (1.5) n = 3143	49 (16) n = 3148	1.1 (0.3) n = 3142	4.9 (1.5) n = 3129	0.6 (0.2) n = 3156
*50–59*	4.9 (1.5) n = 3153	47 (16) n = 3190	1.1 (0.3) n = 3163	4.7 (1.6) n = 3162	0.6 (0.2) n = 3199
*60–69*	4.7 (1.5) n = 3044	44 (15) n = 3080	1.0 (0.3) n = 3065	4.5 (1.5) n = 3068	0.5 (0.2) n = 3082
*70–79*	4.4 (1.4) n = 1414	41 (14) n = 1416	1.0 (0.3) n = 1417	4.2 (1.5) n = 1425	0.5 (0.2) n = 1415
*80* +	4.1 (1.5) n = 268	39 (15) n = 266	0.9 (0.3) n = 267	3.9 (1.5) n = 269	0.5 (0.2) n = 267
*Sex*					
*Male*	5.2 (1.5) n = 5045	50 (16) n = 5096	1.1 (0.3) n = 5078	5.0 (1.6) n = 5044	0.6 (0.2) n = 5150
*Female*	4.5 (1.4) n = 5977	43 (15) n = 6004	1.0 (0.3) n = 5976	4.2 (1.4) n = 6009	0.5 (0.2) n = 5969
Education**					
*Primary*	4.6 (16) n = 2219	43 (1.6) n = 2248	1.0 (0.4) n = 2235	4.4 (1.7) n = 2240	0.5 (0.2) n = 2270
*Secondary*	4.8 (16) n = 2942	46 (1.5) n = 2980	1.1 (0.3) n = 2956	4.7 (1.6) n = 2959	0.5 (0.2) n = 3004
*Tertiary short*	4.9 (16) n = 2237	47 (1.5) n = 2242	1.1 (0.3) n = 2238	4.7 (1.5) n = 2227	0.6 (0.2) n = 2242
*Tertiary long*	4.9 (16) n = 3506	46 (1.4) n = 3510	1.1 (0.3) n = 3506	4.6 (1.5) n = 3508	0.6 (0.2) n = 3482
Income*** (NOK)					
< = *350,000* *(Very low)*	4.4 (1.6) n = 1173	42 (16) n = 1179	1.0 (0.3) n = 1169	4.2 (1.6) n = 1179	0.5 (0.2) n = 1174
*350,000–550,000 (Low)*	4.6 (1.5) n = 2277	44 (16) n = 2304	1.0 (0.3) n = 2290	4.5 (1.6) n = 2284	0.5 (0.2) n = 2311
*550,000–750,000 (Moderate)*	4.8 (1.5) n = 1991	46 (16) n = 2017	1.1 (0.3) n = 2011	4.6 (1.6) n = 2007	0.6 (0.2) n = 2024
*750,000–1,000,000 (High)*	5.0 (1.5) n = 2546	47 (15) n = 2559	1.1 (0.3) n = 2552	4.8 (1.5) n = 2552	0.6 (0.2) n = 2572
*1,000,000* + *(Very high)*	5.0 (1.4) n = 2733	48 (15) n = 2737	1.1 (0.3) n = 2728	4.8 (1.5) n = 2724	0.6 (0.2) n = 2738

GHGE; Greenhouse gas emissions (kg CO_2_-equiv.), TA; Terrestrial acidification (g SO_2_-equiv.), FE; Freshwater eutrophication (g P-equiv.), LU: Land use (m^2^a crop-equiv.), WU; Water use (m^3^), Q1; First quartile (25th percentile), Q3; Third quartile (75th percentile), EI; Energy intake, MJ; Megajoule, NOK; Norwegian Kroners. Values are given as mean (standard deviation), and total number of participants (n) within each category. Where the totals do not sum to the overall number of participants included, the difference reflects the number of participants with unavailable data for those variables. *Tromsø Study, seventh survey, conducted in 2015–16. **Primary (up to 10 years of schooling); secondary education (a minimum of 3 years); tertiary short (college/university less than 4 years); tertiary long (college/university 4 years or more). *** Household’s total taxable income for the previous year

**Fig. 3:**
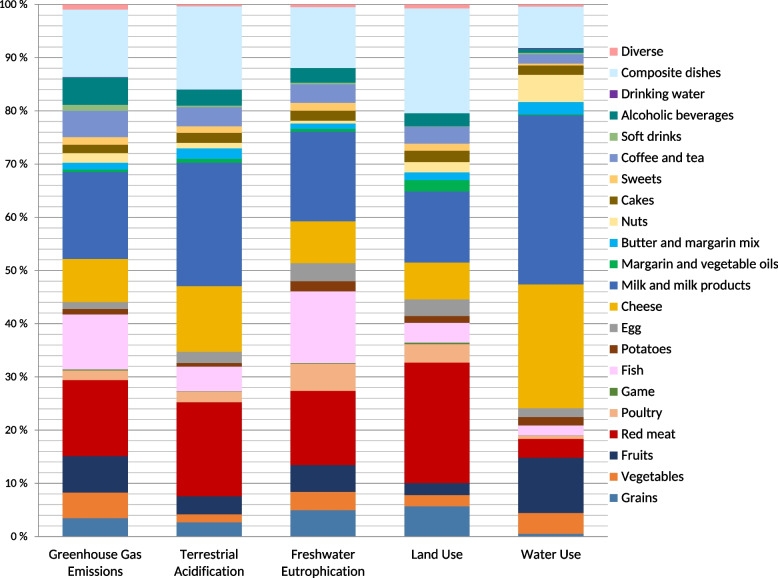
Average contributions from food groups to the daily environmental impact. n varied between 11,022 and 11,119 for the different analyses. The “Diverse” group includes snacks and sauces. The “Composite dishes” group includes pizza, pie, lasagna, kebab, wrap, taco, lapskaus (meat and vegetable stew), minced meat stew, Spanish chicken stew, spring rolls, wok, hamburger, lamb and cabbage stew, and rice porridge. The “Sweets” group includes sugar, honey, chocolate, jam, and candy. The “Cakes” group includes pastry, buns, waffle, cookies, cakes, and muffins

In the typical Northern Norwegian diet, milk and milk products were the highest contributors to the environmental impact, accounting for 16% of the GHGE, 23% of terrestrial acidification, 17% of freshwater eutrophication, 13% of land use, and 31% of water use (Fig. 3). Red meat accounted for 14% of the GHGE, 17% of terrestrial acidification, 14% of freshwater eutrophication, and 22% of land use, with a lower impact on water use (3%). Cheese significantly affected water use, contributing 23%. We could not divide the composite dishes into their ingredients, which led to this group making a notable contribution to the environmental impact and underestimating other food groups. A detailed table is provided in S2.

### Variations in environmental impact by different sociodemographic groups

Significant differences were observed in most environmental impact categories according to sex, age, and BMI in unadjusted analyses (S3, S4). Men had a higher impact than women, younger adults had a higher impact than older participants, and individuals with obesity had a higher impact than those without obesity. To ensure the robustness of our findings, we conducted a sensitivity analysis excluding individuals classified as underweight. This exclusion did not substantially alter the results (data not shown). Analyses on education and income levels yielded mixed results. Differences between primary and tertiary education levels were statistically significant for GHGE, freshwater eutrophication, and water use. Only the differences between very low/low and high/very high-income levels showed statistical significance for the GHGE and freshwater eutrophication impact categories.

### Differential associations of sociodemographic factors and their interactions on environmental impact

All the multivariate regression models explained a substantial proportion of the variability in the impact categories, ranging from 53% to 71%, with generally weak effect estimates (S5). After adjustments, sex was not significantly associated with any of the environmental impact categories, while age was inversely associated with all, except for 50–69-year-olds, who displayed a positive association with GHGE. The highest educational level was inversely associated with the GHGE and land use. Moderate to very high-income levels were positively associated with GHGE, freshwater eutrophication, and land use. BMI was positively associated with almost all environmental impact categories. Exclusion of individuals who were underweight did not substantially change the results (data not shown).

**Fig. 4:**
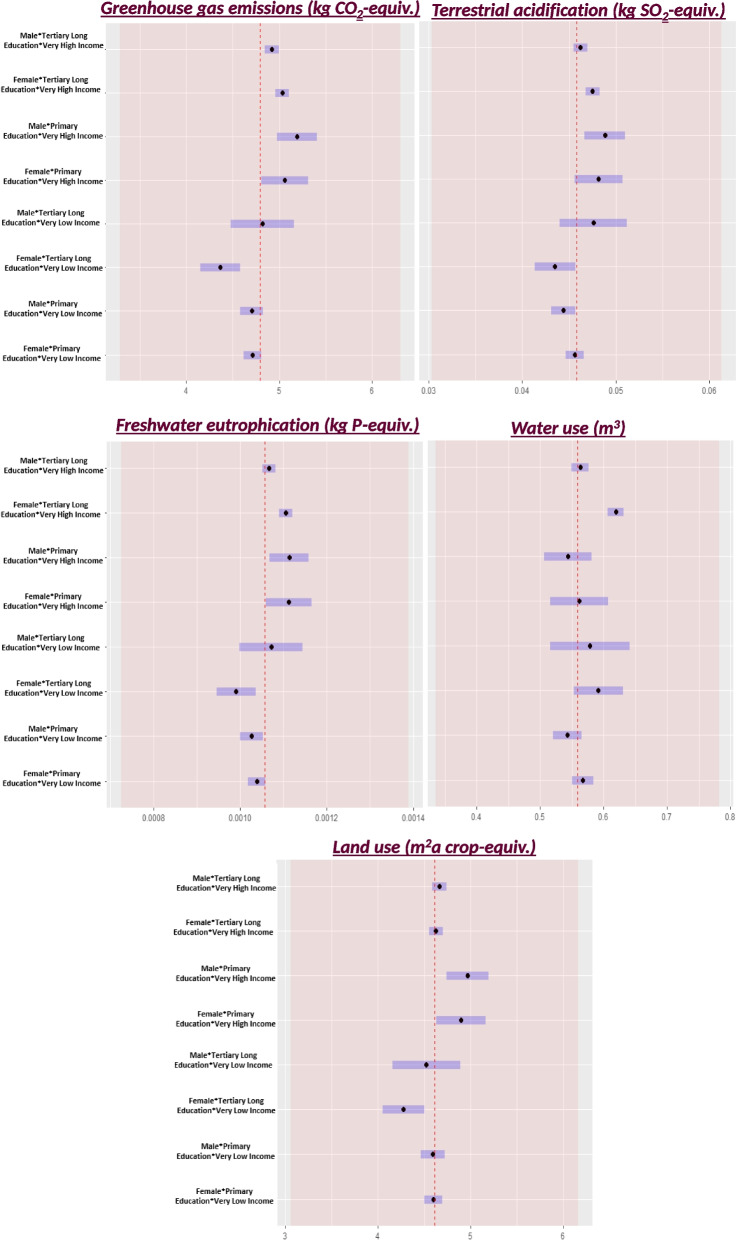
Marginal means with confidence intervals for each interaction category of interest. The dashed red lines represent the mean of the whole population, while the red shaded area indicates the standard deviation of the whole population mean

Across all environmental impact categories, the inclusion of three-way interactions between sex, education, and income did not significantly improve the adjusted model, indicating that it did not explain more of the variation in the data. Of the estimated marginal means in the interaction analyses (Fig. 4), the differences between males and females were minimal. Females showed higher marginal means for water use. Participants with higher education generally had lower means for most environmental indicators while higher-income individuals generally exhibit higher means across all impact categories regardless of sex or education. Despite these trends, most pairwise comparisons yielded non-significant associations and the magnitude of the differences between many groups were relatively minor (S6, S7). Several estimates exhibited considerable uncertainty as reflected by wide confidence intervals, suggesting precision issues with these estimates.

## Discussion

In this study, the mean impact of food consumption in Northern Norway in terms of CO_2_-eq was estimated to be 4.8 kg per person per day. This corresponds to the estimations in a recent study by Lengle et al. [38] (4.7 kg CO_2_-eq per person per day), which used the same environmental impact database with a different dietary assessment method focusing on Norwegian diets without a specific regional emphasis on the North. This estimate is 2.5 times higher than the planetary boundary target for GHGE, as downscaled from the EAT-Lancet Commission’s targets using an equal per capita approach [38, 61]. Most previous studies on the environmental sustainability of food consumption have focused primarily on GHGE, with only a few examining Norwegian diets [5, 6, 26, 27, 38, 59, 61–66]. Reported GHGE estimates for Norwegian diets range between ∼ 3.8 and 4.9 kg CO₂-eq per person per day, likely reflecting methodological differences, especially in how environmental values are calculated. According to the study by Alves et al. [67] analyzing ten European countries, reported mean GHGE ranged from 4.0 kg CO₂-eq/day in Spain to 6.5 kg CO₂-eq/day in France. Lengle et al. [38] also assessed the remaining impact categories we explored, and our estimates are similar (49 vs. 50 g SO_2_-eq, 1.1 vs. 1 g P-eq, 5.3 vs. 4.6 m^2^a, and 0.5 vs. 0.6 m^3^, respectively for terrestrial acidification, freshwater eutrophication, land use, and water use).

The contribution of plant-based foods to the various environmental impact categories was relatively low compared to that of animal-based foods. Milk and milk products were the greatest contributors to GHGE, terrestrial acidification, freshwater eutrophication, and land use, followed by red meat. This aligns with the findings in similar studies conducted in Scandinavia, although in some, red meat is the leading contributor [26, 38, 68]. Cheese and fruit are important contributors to water use, in addition to milk and milk products. Globally, country-level cumulative environmental impacts stem mainly from land-based food production [69]. However, with Norway’s extensive coastline and fisheries and aquaculture industries, 88% of the environmental impact is ocean-based [69]. Although a large proportion of this production is exported [69], fish remains a significant contributor to the environmental impact of dietary consumption in Northern Norway. This study showed higher contributions from fish consumption across most indicators, approximately two to seven times greater than the findings of Lengle et al. [38], reflecting differences in regional consumption patterns. In addition, although Northern Norway has relatively higher game consumption than other regions, the contribution from their consumption as a separate food group was not high [39]. Composite dishes appear to be an important contributor across all impact categories, as they cannot be broken down into their ingredients. Considering their varied contents, their impact will likely spread across food groups.

Regarding the population subgroups, the environmental impact varied significantly across different intrapersonal factors. Without adjusting the models, men, younger adults, and people with obesity appeared to have a higher environmental impact, whereas education and income levels displayed mixed results. However, the significance of sex differences diminishes when controlling for energy intake, physical activity, and smoking status, emphasizing the importance of these confounders, as mentioned in other studies [27]. This contrasts with the findings of a study by Lengle et al. [38], who reported that men had significantly higher dietary environmental impacts than women across GHGE, terrestrial acidification, freshwater eutrophication, and land use, even after energy adjustments. However, their mean differences range between 1% and 7%, which may not be meaningful from a public health perspective. Therefore, although our results appear different, this contrast is less substantial than initially thought. When considering other European countries, females generally exhibit lower environmental impact [67, 70]. However, similar to our results, in Greece and Spain there were no significant differences between males and females, as reported by Alves et al. [67]. The association between the environmental impact and education showed inconsistent results among European countries [67]. In our adjusted models, individuals with the highest educational level had a lower impact on GHGE and land use. By contrast, Lengle et al. [38] reported that low-to-moderate educational attainment was associated with a lower impact on freshwater eutrophication and water use after energy adjustment. However, the mean differences they reported were small (up to 1%), and the present study adjusted for smoking and physical activity, which might account for certain of these differences. Both education and income are highlighted in several previous studies as key indicators for dietary environmental impacts, with mostly lower education being associated with higher impacts and higher income levels being associated with higher impacts through an easy reach to meat, fish, and dairy products [48, 61, 71–74]. Our findings align with this, particularly regarding the association among education level, GHGE, and land use. Additionally, the adjusted models revealed that individuals with moderate-to-very-high income levels exhibited higher impacts on GHGE, freshwater eutrophication, and land use, further supporting the established patterns. Controlling for energy intake, while age was inversely associated, people with higher BMI levels had a higher environmental impact across all categories, which means that their impact is not only influenced by high energy intake but also choice of food. These findings were generally aligning with findings from other European countries [25, 67, 70, 75].

Contrary to our theorizing, the combined influence of the interactions between sex, education, and income factors did not significantly lead to a greater impact on the environmental impact categories beyond what can be explained by considering each variable separately. Although pairwise comparisons revealed a few significant interactions, all associations were weak, and the marginal means for the interaction categories closely aligned with the total population means for each environmental impact category. Most results fell within the standard deviations of these population means, indicating that while sex, education, and income interactions introduced some variability, the environmental impacts for most groups remained consistent with broader population trends. This lack of distinct patterns of disadvantage points to the absence of substantial differences across the intersectional categories analyzed. Only minor variations were observed, as expected in diverse populations. Although this could reflect true homogeneity, it might be influenced by data limitations or analytical methods. Reliance solely on *p*-values is insufficient, and weak effect estimates further complicate the interpretation. Therefore, while these findings suggest potential equality across the analyzed intersectional categories in the study population, caution is warranted when drawing conclusions.

### Strengths and limitations

This study has several strengths. To our knowledge, this is the first study to estimate the environmental impact of dietary consumption among the population living in Northern Norway and the first to use an intersectional approach in this assessment, specifically examining the interplay between sex, education, and income.

This study used a large sample from an ongoing population-based cohort of adult and elderly women and men from urban and rural living areas in Northern Norway, with relatively high overall participation. A previously validated comprehensive FFQ with a minimum completeness rate of 90% was used.

The environmental impact database used in this study is highly comprehensive and contains information on five different impact categories. The database offers the current best geographical proximity for our study, as it is based on LCA data for food items representing the Norwegian habitual diet and food market. All relevant literature was assessed for quality, and those published before 2010 were excluded [58].

Furthermore, an intersectional perspective was acquired, opening the path to enrich our understanding of the complex interplay between various factors and recognizing that many important inequalities are intersectional. Within this framework, our pre-chosen categories of interest (sex, education, and income) serve as another strength, as the literature supports that “although context motivates the variability seen in the factors that contribute to within-country inequality, there are certain consistently marginalized groups: women, those without education, Indigenous Peoples, and the poor” [41, 46]. The literature highlights the importance of mainstreaming intersectionality considerations into research [41, 46], and this study, we believe, is an important addition.

However, this study had important limitations. A general limitation of the FFQ is the risk of misclassification due to recall or social desirability bias, implying that some participants might overreport or underreport their actual dietary intake. In addition, we were not able to express income on a per-person basis due to data unavailability. Income reported by large households may not accurately reflect the income available to each individual within the household.

Further, the study sample consisted of people aged ≥ 40 years, which can affect the representativeness of the contribution from food groups. For example, younger people might have been drinking more soft drinks than older people. Further, non-respondents often differ from respondents in various characteristics in population-based studies [76, 77]. It is indicated that non-respondents are typically men, less educated, and have lower economic status in Tromsø7 survey [78]. The inclusion criterion of considering only participants who completed 90% or more of the FFQ may have contributed to selection bias.

Another limitation was that avoidable food waste at the retail and household levels was not consistently accounted for in the impact estimates, as LCA data were not available for some. This may have led to an underestimation of the dietary environmental impact.

In our study, outliers that varied across the datasets for different environmental impact categories led to the formation of different subgroups after exclusion. While this may seem confusing, it is important to note that the nature of each impact category is distinct. They could not be directly compared or combined. Therefore, because each impact category should be interpreted independently, the existence of different samples across categories reflects the unique characteristics of each dataset. However, the IQR method used to remove outliers is not immune to criticism. The classification of outliers relies on statistical thresholds rather than on an inherent understanding of whether the data points genuinely reflect atypical consumption or are part of a broader but less common pattern. However, in line with established practices, including the common use of box plots, our analysis emphasizes understanding the broader or typical distribution of consumption patterns that tend to have the most significant and widespread environmental impacts. In this analysis, the exclusions made using this method resulted in the removal of less than 4% of the total sample across all categories, making it unlikely to affect the integrity and representativeness of the dataset. This minor adjustment aligns with the rationale used in statistical significance testing in the literature, where the commonly accepted 5% alpha level acknowledges a tolerable risk of false positives [79]. However, as even modest data exclusion could potentially influence the findings, careful consideration should be given to their possible effects on the overall analysis.

This study has certain limitations concerning the intersectionality component. Despite the relatively large total sample size, each intersectional group had a smaller sample range of 24–850 participants (S8), which may have limited the statistical power. Small subgroup sizes and the complexity of intersectional factors can affect the ability to detect significant differences.

The literature highlights that insufficient data exist to characterize intersectionality consistently [41]. Accordingly, this study faces a common limitation in many quantitative intersectionality studies: the provisional use of broad categories for sex, education, and income, largely because of the lack of a gold standard. From an intersectional perspective, these categories should be viewed as part of socially constructed power dynamics, and research should focus on how they reflect underlying issues such as systemic inequality and how they interact to produce combined effects. Our theorization and available datasets may not fully capture the complexities of these interactions.

Another limitation was the absence of ethnicity in our intersectional framework. While playing a crucial role in social power dynamics, ethnicity may influence food consumption patterns and environmental impacts through cultural preferences. Given that our Arctic population includes various minority and Indigenous groups, integrating ethnicity would have significantly enriched the research. We could not incorporate this important dimension into our analysis due to the lack of available data.

Despite limitations, this study demonstrates the potential for applying an intersectional framework to environmental research while underscoring the need for more comprehensive and nuanced data to accurately capture these complex dynamics. Strengthening food-based dietary guidelines with these dynamics to integrate health and sustainability considerations is crucial, especially in Norway, where current national guidelines focus solely on diet-health connections without addressing environmental impacts [80]. Evidence-based policymaking should establish shared goals for sustainable food consumption through collaboration with all relevant stakeholders, balancing diverse interests and objectives [37, 61, 80, 81]. An enabling environment to encourage healthy and sustainable food choices can be fostered through education and increased awareness, economic incentives, marketing strategies (e.g., labeling, product placement), national regulations, and regional priorities, while ensuring a just transition accounting for the unique realities of specific contexts by supporting targeted interventions [37, 61, 80, 81].

## Conclusions

This study is the first to assess the environmental impact of dietary consumption in Northern Norway, utilizing an intersectional approach to provide a more comprehensive insight. The environmental burden of the Northern Norwegian diet remains high, with dairy products being the largest contributor. Age was inversely associated with environmental impacts, while BMI showed a positive association. Sex did not influence environmental burden, and the results for education and income varied. We observed no significant multiplicative effect of sex, education, and income, explaining more of the variance, which may suggest general potential equality across our intersectional groups. Further research with larger samples and refined methodologies may be necessary to deepen our understanding of these dynamics and explore whether the observed patterns persist under different conditions or with alternative analytical approaches. Policies and interventions should aim to reduce the environmental impact arising from food consumption while also incorporating an intersectional approach that can help develop tailored strategies to more effectively address the diverse contributions to environmental impact across different demographic groups.

## Supplementary Information

Supplementary Material 1

## Data Availability

The datasets generated and/or analyzed during the current study are not publicly available as it involves sensitive personal data.

## Abbreviations

ANOVA: Analysis of Variance
BMI: Body Mass Index
CO_2_: Carbon Dioxide
D: Day
DASH: Dietary Approaches to Stop Hypertension
EI: Energy Intake
EQ/EQUIV: Equivalent
FAO: Food and Agriculture Organization of the United Nations
FFQ: Food Frequency Questionnaire
G: Gram
GHGE: Greenhouse Gas Emissions
HEI: Healthy Eating Index
IQR: Interquartile Range
KBS: Food Composition and Nutrient Calculation System
KG: Kilogram
KJ: Kilojoule
LCA: Life Cycle Analysis
M: Meter
MJ: Megajoule
NOK: Norwegian Krone
P: Phosphorus
Q: Quartile
SO_2_: Sulfur Dioxide
STROBE: Strengthening the Reporting of Observational Studies in Epidemiology
UiO: University of Oslo
WHO: World Health Organization
