# Supplementary material for: Environmental impact of food consumption and sociodemographic factors in Northern Norway through an intersectional lens: a cross-sectional study
Source: BMC Public Health. 2025 Aug 30;25:2978. doi: 10.1186/s12889-025-23899-3 (PMC12398146; doi:10.1186/s12889-025-23899-3)
Supplement: Supplementary file 1 — Supplementary Material 1 [file 12889_2025_23899_MOESM1_ESM.pdf]

Supplementary Materials

S1. Participant Characteristics

	FFQ Completed N=15,138	***FFQ >=90.0% N=11,449***	***FFQ <90.0% N=3,689***
	**% (n)**	**% (n)**	**% (n)**
		Age groups*	
40-49	25.7 (3896)	28.6 (3278)	16.8 (618)
50-59	27.3 (4116)	28.8 (3300)	22.1 (816)
60-69	28.2 (4274)	27.6 (3152)	30.4 (1122)
70-79	15.2 (2301)	12.6 (1447)	23.1 (854)
80+	3.6 (551)	2.4 (272)	7.6 (279)
		Sex*	
Male	46.1 (6985)	46.6 (5337)	44.7 (1648)
Female	53.9 (8153)	53.4 (6112)	55.3 (2041)
		Body Mass Index	
Normal <=24.99	32.9 (4966)	33.3 (3800)	31.7 (1166)
Overweight 25.0 - 29.99	43.8 (6610)	43.6 (4986)	44.2 (1624)
Obese >=30.00	23.3 (3522)	23.1 (2636)	24.1 (886)
		Smoking Status*	
Current smoker	12.6 (1892)	12.6 (1431)	12.7 (461)
Former smoker	45.6 (6845)	44.9 (5104)	47.9 (1741)
Non-smoker	41.8 (6264)	42.5 (4834)	39.4 (1430)
		Physical Activity Level[a]*	
Sedentary	13.6 (1996)	13.2 (1476)	15.0 (520)
Light	58.7 (8598)	58.6 (6552)	58.9 (2046)
Moderate	24.8 (3640)	25.2 (2815)	23.7 (825)
Vigorous	2.9 (423)	3.0 (339)	2.4 (84)
		Educational Level[b]*	
Primary	23.7 (3533)	20.6 (2333)	33.8 (1200)
Secondary	27.2 (4056)	27.4 (3098)	26.9 (958)
Tertiary short	19.4 (2880)	20.3 (2305)	16.2 (575)
Tertiary long	29.7 (4414)	31.7 (3591)	23.1 (823)
		Income Level (NOK)*	
<=350000 (Very low)	13.5 (1957)	11.0 (1220)	21.8 (737)
351000 – 550000 (Low)	21.6 (3138)	21.3 (2372)	22.6 (766)
551000 – 750000 (Moderate)	18.4 (2678)	18.8 (2090)	17.4 (588)
750000 – 1000000 (High)	22.7 (3289)	23.7 (2644)	19.0 (645)
1000000 + (Very high)	23.8 (3460)	25.2 (2809)	19.2 (651)

a Exercise and physical activity in leisure time over the last year: sedentary (reading, watching TV/screen or other sedentary activity); light (walking, cycling or other forms of exercise at least 4 h a week); moderate (participation in recreational sports, heavy gardening, snow shoveling, etc., at least 4 h a week); vigorous (participation in hard training or sports competitions, regularly, several times a week).
b Primary (up to 10 years of schooling); secondary education (a minimum of 3 years); tertiary short (college/university less than 4 years); tertiary long (college/university 4 years or more).
NOK; Norwegian kroners
**A statistically significant difference was observed (p<0.05) between the groups FFQ >= 90.0% and FFQ<90.0% tested using Pearson's chi-square test.*
Where the totals do not sum to the overall number of participants, the difference reflects the number of participants with unavailable data for those variables.

S2. Absolute values of contribution of the 22 different food groups to each impact category

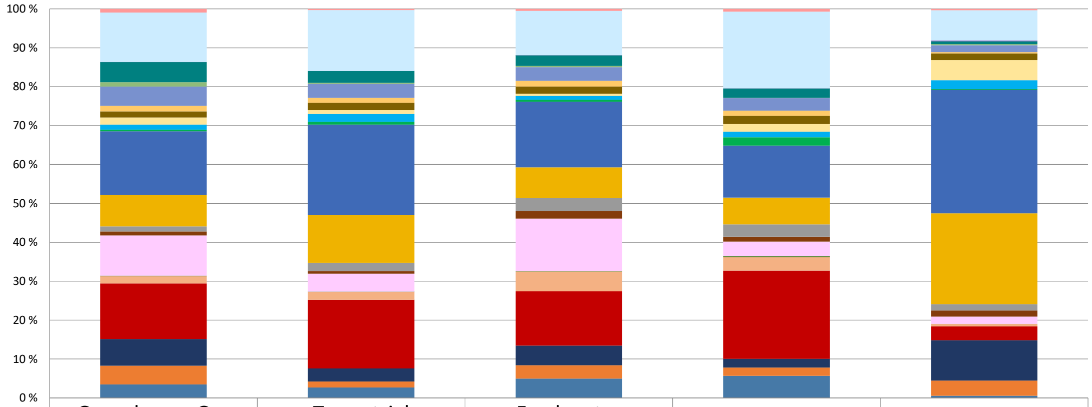

	Greenhouse Gas Emissions	Terrestrial Acidification	Freshwater Eutrophication	Land Use	Water Use
Diverse	0,037979006	1,32E-04	4,68E-06	2,90E-02	0,001842415
Composite dishes	0,514090842	6,30E-03	1,07E-04	7,99E-01	0,03925436
Drinking water	0,002335582	4,11E-06	8,53E-08	4,24E-05	0,000818617
Alcoholic beverages	0,208946304	1,23E-03	2,57E-05	9,91E-02	0,003704688
Soft drinks	0,045082261	1,12E-04	2,83E-06	2,60E-03	0,001442482
Coffee and tea	0,200480446	1,45E-03	3,28E-05	1,29E-01	0,008882024
Sweets	0,057200419	5,02E-04	1,37E-05	5,46E-02	0,001724259
Cakes	0,064423289	7,62E-04	1,74E-05	8,62E-02	0,008796778
Nuts	0,07320187	4,08E-04	5,24E-06	7,78E-02	0,02590367
Butter and margarin mix	0,051968212	8,05E-04	9,32E-06	5,98E-02	0,01147011
Margarin and vegetable oils	0,018627728	2,80E-04	4,87E-06	8,61E-02	0,000964492
Milk and milk products	0,66124279	9,36E-03	1,58E-04	5,42E-01	0,160154268
Cheese	0,327528685	4,97E-03	7,37E-05	2,79E-01	0,117467632
Egg	0,053881485	8,50E-04	3,16E-05	1,28E-01	0,008137484
Potatoes	0,040175698	2,80E-04	1,79E-05	5,21E-02	0,008045076
Fish	0,418857747	1,86E-03	1,26E-04	1,50E-01	0,009417603
Game	0,007123216	3,01E-05	1,42E-06	1,28E-02	0,000185038
Poultry	0,074530596	8,00E-04	4,76E-05	1,40E-01	0,00309463
Red meat	0,578546546	7,12E-03	1,31E-04	9,18E-01	0,017800469
Fruits	0,277377518	1,37E-03	4,71E-05	9,11E-02	0,052308084
Vegetables	0,1947499	6,14E-04	3,20E-05	8,54E-02	0,019735944
Grains	0,140040293	1,08E-03	4,65E-05	2,30E-01	0,002729957

Greenhouse gas emissions (kg CO2-equivalent), terrestrial acidification (g SO2-equivalent), freshwater eutrophication (g P-equivalent), land use (m²a crop-equivalent), and water use (m³)

S3. Socio-demographic categories stratified by quartiles of the total environmental impact across all the environmental impact categories

Indicator		**Greenhouse Gas Emissions**		
Quartiles	**Q1**	**Q2**	**Q3**	**Q4**
Age				
40-49	23.6% (651)	27.1% (747)	30.0% (826)	33.3% (919)
50-59	25.5% (703)	28.1% (773)	29.1% (803)	31.7% (874)
60-69	29.2% (804)	28.9% (796)	27.1% (746)	25.3% (698)
70-79	17.4% (480)	13.5% (372)	12.1% (334)	8.3% (228)
80+	4.3% (118)	2.4% (67)	1.7% (46)	1.3% (37)
Sex				
Female	68.7% (1892)	60.5% (1668)	48.6% (1339)	39.1% (1078)
Male	31.3% (864)	39.5% (1087)	51.4% (1416)	60.9% (1678)
Education				
Primary	27.5% (748)	18.8% (512)	17.3% (471)	17.8% (488)
Secondary	26.9% (732)	26.1% (711)	26.5% (724)	28.3% (775)
Tertiary short	17.8% (483)	21.3% (580)	22.1% (604)	20.8% (570)
Tertiary long	27.8% (757)	33.8% (920)	34.0% (928)	33.0% (901)
Income				
<=350000	16.9% (447)	10.4% (279)	8.7% (234)	7.9% (213)
351000-550000	26.4% (699)	20.8% (557)	18.5% (498)	19.4% (523)
551000-750000	18.4% (486)	19.8% (532)	18.0% (484)	18.1% (489)
750000-1000000	20.2% (534)	22.9% (615)	26.0% (699)	25.8% (698)
1000000+	18.1% (480)	26.1% (700)	28.8% (774)	28.8% (779)
Body Mass Index				
Normal<=24.99	35.5% (977)	35.5% (975)	34.0% (933)	29.1% (800)
Overweight 25.0-29.99	42.3% (1164)	42.6% (1171)	44.4% (1220)	45.5% (1252)
Obese>=30.00	22.1% (608)	22.0% (604)	21.6% (593)	25.4% (699)
Indicator		**Terrestrial Acidification**		
Quartiles	**Q1**	**Q2**	**Q3**	**Q4**
Age				
40-49	19.7% (546)	27.7% (714)	31.5% (873)	36.6% (1015)
50-59	26.5% (736)	28.5% (792)	29.4% (817)	30.5% (845)
60-69	31.8% (883)	29.3% (812)	26.3% (729)	23.6% (656)
70-79	17.8% (495)	13.9% (387)	11.4% (316)	7.9% (218)
80+	4.1% (115)	2.5% (70)	1.4% (40)	1.5% (41)
Sex				
Female	67.8% (1881)	60.1% (1668)	49.7% (1380)	38.7% (1075)
Male	32.2% (894)	39.9% (1107)	50.3% (1395)	61.3% (1700)
Education				
Primary	27.4% (748)	19.8% (544)	16.8% (461)	18.0% (495)
Secondary	26.3% (718)	26.7% (735)	27.3% (750)	28.2% (777)
Tertiary short	17.8% (485)	20.3% (557)	23.2% (638)	20.4% (562)
Tertiary long	28.6% (780)	33.2% (914)	32.7% (899)	33.3% (917)
Income				
<=350000	16.1% (430)	10.3% (279)	9.5% (257)	7.8% (213)
351000-550000	25.7% (684)	21.0% (566)	19.1% (516)	19.7% (538)
551000-750000	18.9% (502)	20.1% (543)	18.2% (493)	17.6% (479)
750000-1000000	20.3% (541)	22.7% (614)	26.3% (710)	25.4% (694)
1000000+	19.0% (506)	25.9% (699)	26.9% (727)	29.5% (805)
Body Mass Index				
Normal<=24.99	35.8% (992)	34.8% (963)	32.7% (906)	30.8% (853)
Overweight 25.0-29.99	42.6% (1179)	43.2% (1196)	44.4% (1229)	44.5% (1233)
Obese>=30.00	21.6% (597)	22.0% (608)	22.9% (635)	24.7% (685)
Indicator		**Freshwater Eutrophication**		
Quartiles	**Q1**	**Q2**	**Q3**	**Q4**

Age				
40-49	22.1% (610)	26.9% (742)	30.7% (849)	34.0% (941)
50-59	25.0% (691)	29.0% (801)	28.7% (793)	31.8% (878)
60-69	30.2% (834)	29.5% (815)	26.4% (729)	24.9% (687)
70-79	18.2% (503)	12.4% (343)	12.6% (348)	8.1% (223)
80+	4.6% (126)	2.2% (62)	1.6% (44)	1.3% (35)
Sex				
Female	68.8% (1902)	59.4% (1641)	49.6% (1370)	38.5% (1063)
Male	31.2% (862)	40.6% (1122)	50.4% (1393)	61.5% (1701)
Education				
Primary	27.8% (758)	19.1% (523)	17.2% (471)	17.6% (483)
Secondary	26.2% (715)	27.1% (740)	25.8% (705)	29.1% (796)
Tertiary short	17.6% (479)	20.2% (553)	23.4% (640)	20.7% (566)
Tertiary long	28.4% (773)	33.6% (918)	33.6% (921)	32.6% (894)
Income				
<=350000	16.6% (438)	10.1% (272)	9.2% (247)	7.8% (212)
351000-550000	25.7% (679)	21.4% (578)	19.1% (514)	19.1% (519)
551000-750000	18.8% (495)	19.5% (526)	18.6% (502)	18.0% (488)
750000-1000000	20.3% (535)	23.4% (633)	24.7% (665)	26.5% (719)
1000000+	18.7% (493)	25.7% (694)	28.4% (765)	28.6% (776)
Body Mass Index				
Normal<=24.99	36.1% (995)	35.1% (969)	33.6% (927)	29.1% (802)
Overweight 25.0-29.99	42.2% (1163)	43.3% (1194)	44.4% (1222)	45.1% (1245)
Obese>=30.00	21.7% (598)	21.6% (595)	22.0% (606)	25.8% (713)
Indicator		**Land Use**		
Quartiles	**Q1**	**Q2**	**Q3**	**Q4**
Age				
40-49	20.7% (571)	27.7% (764)	30.6% (845)	34.3% (949)
50-59	26.5% (732)	27.9% (772)	29.3% (809)	30.7% (849)
60-69	31.0% (856)	28.2% (778)	27.1% (749)	24.8% (685)
70-79	17.4% (482)	14.0% (387)	11.4% (316)	8.7% (240)
80+	4.5% (123)	2.2% (62)	1.6% (44)	1.4% (40)
Sex				
Female	69.9% (1933)	61.7% (1706)	48.6% (1343)	37.2% (1027)
Male	30.1% (831)	38.3% (1057)	51.4% (1420)	62.8% (1736)
Education				
Primary	26.0% (709)	18.7% (511)	18.0% (493)	19.2% (527)
Secondary	25.0% (681)	26.5% (724)	26.7% (732)	30.0% (882)
Tertiary short	18.2% (498)	20.9% (571)	21.1% (578)	21.2% (580)
Tertiary long	30.8% (841)	33.8% (922)	34.2% (936)	29.5% (809)
Income				
<=350000	16.3% (431)	10.1% (272)	9.1% (245)	8.5% (231)
351000-550000	25.4% (672)	21.2% (572)	19.0% (511)	19.5% (529)
551000-750000	18.8% (497)	18.4% (496)	18.8% (505)	18.7% (509)
750000-1000000	20.3% (539)	22.8% (614)	25.7% (691)	26.1% (708)
1000000+	19.3% (511)	27.5% (740)	27.3% (734)	27.2% (739)
Body Mass Index				
Normal<=24.99	37.3% (1027)	36.3% (1000)	32.2% (889)	28.6% (789)
Overweight 25.0-29.99	42.2% (1163)	42.8% (1178)	45.4% (1253)	44.4% (1223)
Obese>=30.00	20.6% (567)	20.9% (577)	22.4% (617)	27.0% (745)
Indicator		**Water Use**		
Quartiles	**Q1**	**Q2**	**Q3**	**Q4**
Age				
40-49	21.4% (596)	25.7% (714)	29.5% (819)	36.9% (1027)
50-59	27.5% (764)	28.6% (794)	29.2% (811)	29.9% (830)
60-69	31.3% (869)	29.7% (826)	26.9% (747)	23.0% (640)
70-79	16.8% (466)	13.2% (366)	12.6% (350)	8.4% (233)
80+	3.1% (85)	2.9% (80)	1.9% (52)	1.8% (50)

Sex				
Female	61.1% (1699)	56.5% (1570)	51.7% (1438)	45.4% (1262)
Male	38.9% (1081)	43.5% (1210)	48.3% (1341)	54.6% (1518)
Education				
Primary	27.4% (750)	21.3% (585)	17.7% (486)	16.3% (449)
Secondary	29.4% (804)	27.7% (762)	26.4% (727)	25.8% (711)
Tertiary short	19.1% (523)	19.8% (544)	22.1% (609)	20.5% (566)
Tertiary long	24.1% (661)	31.3% (861)	33.8% (931)	37.4% (1029)
Income				
<=350000	14.7% (392)	11.0% (298)	9.2% (250)	8.6% (234)
351000-550000	23.7% (635)	21.6% (584)	20.4% (557)	19.7% (535)
551000-750000	19.5% (521)	18.8% (508)	18.8% (512)	17.8% (483)
750000-1000000	21.9% (587)	23.9% (646)	25.0% (680)	24.3% (659)
1000000+	20.2% (540)	24.7% (667)	26.6% (725)	29.7% (806)
Body Mass Index				
Normal<=24.99	31.7% (879)	32.0% (886)	34.3% (952)	35.6% (980)
Overweight 25.0-29.99	43.3% (1200)	45.4% (1259)	43.7% (1211)	42.4% (1177)
Obese>=30.00	25.1% (695)	22.6% (627)	22.0% (611)	22.0% (610)

S4. ANOVA Results

	Df	Sum Sq	Mean Sq	F value	P value	Tukey correction (adjusted p value)
**OUTCOME: TOTAL TERRESTRIAL ACIDIFICATION ARISING FROM FOOD CONSUMPTION**						
**SEX**	1	0.12850	0.128501	589.9792	< 2.2e-16*	Significant
**AGE**	4	0.09662	0.024154	110.8977	< 2.2e-16*	Only group 70-79 and 80+ is not significant (0.3444230)
**EDUCATION**	3	0.00157	0.000525	2.4094	0.065026	Not significant
**INCOME**	4	0.00166	0.000414	1.9022	0.107083	Not significant
**BMI**	2	0.00235	0.001174	5.3889	0.004579*	Only group normal weight-overweight is not significant (0.6767049)
**RESIDUALS**	10691	2.32856	0.000218			
**OUTCOME: TOTAL FRESHWATER EUTROPHICATION ARISING FROM FOOD CONSUMPTION**						
**SEX**	1	0.00006036	6.0361e-05	600.2461	< 2.2e-16*	Significant
**AGE**	4	0.00003531	8.8260e-06	87.7728	< 2.2e-16*	Significant
**EDUCATION**	3	0.00000190	6.3200e-07	6.2858	0.0002948*	Groups secondary-tertiary short, secondary-tertiary long, and tertiary short-tertiary long are not significant (0.9283668, 0.9800789, 0.9926664)
**INCOME**	4	0.00000190	4.7400e-07	4.7177	0.0008389*	Only groups very low income-high income and very low income-very high income are significant (0.0303665, 0.0056028)
**BMI**	2	0.00000148	7.4200e-07	7.3783	0.0006279*	Only group normal weight-overweight is not significant (0.3715594)
**RESIDUALS**	10645	0.00107047	1.0100e-07			
**OUTCOME: TOTAL GREENHOUSE GAS EMISSIONS ARISING FROM FOOD CONSUMPTION**						
**SEX**	1	1241.9	1241.94	593.1228	< 2.2e-16*	Significant
**AGE**	4	556.5	139.13	66.4445	< 2.2e-16*	Only group 40-49 and 50-59 is not significant (0.1216089)
**EDUCATION**	3	27.1	9.04	4.3166	0.004763*	Only groups primary-tertiary short, and primary-tertiary long are significant (0.0096325, 0.0161837)
**INCOME**	4	67.8	16.94	8.0907	1.648e-06*	Only groups very low income-high income, very low income-very high income and low income-very high income are significant (0.0076769, 0.0006175, 0.0072432)
**BMI**	2	28.1	14.03	6.6988	0.001238*	Only group normal weight-overweight is not significant (0.4502983)
**RESIDUALS**	10615	22226.7	2.09			
**OUTCOME: TOTAL LAND USE ARISING FROM FOOD CONSUMPTION**						
**SEX**	1	1653.8	1653.83	763.8489	< 2.2e-16*	Significant
**AGE**	4	775.0	193.74	89.4830	< 2.2e-16*	Significant
**EDUCATION**	3	40.6	13.54	6.2525	0.0003092*	Only group secondary-tertiary long is significant (0.0001255)
**INCOME**	4	25.1	6.28	2.9021	0.0205597*	Not significant
**BMI**	2	78.0	39.01	18.0185	1.541e-08*	Only group normal weight-overweight is not significant (0.0816517)
**RESIDUALS**	10641	23039.1	2.17			
**OUTCOME: TOTAL WATER USE ARISING FROM FOOD CONSUMPTION**						
**SEX**	1	6.81	6.8106	143.1817	< 2e-16*	Significant
**AGE**	4	13.54	3.3858	71.1812	< 2e-16*	Only groups 60-69 and 80+, and 70-79 and 80+ are not significant (0.2564848, 0.9999655)
**EDUCATION**	3	4.78	1.5940	33.5120	< 2e-16*	Only group secondary-tertiary short is not significant (0.2602209)
**INCOME**	4	0.10	0.0248	0.5217	0.71983	Not significant
**BMI**	2	0.74	0.3690	7.7574	0.00043*	Only group overweight-obese is not significant (0.7572824)
**RESIDUALS**	10714	509.62	0.0476			
**ANAVO; Analysis of Variance, DF; Degree of Freedom, *Statistically significant, p<0.05**						

S5. Regression Results

	ESTIMATE	STANDARD ERROR	T STATISTICS	P VALUE
**OUTCOME: TOTAL TERRESTRIAL ACIDIFICATION ARISING FROM FOOD CONSUMPTION[A]**				
**SEX**	-1.202e-03	7.909e-04	-1.520	0.12853
**AGE**				
**50-59**	-1.238e-03	2.251e-04	-5.500	3.88e-08*
**60-69**	-1.852e-03	2.390e-04	-7.747	1.03e-14*
**70-79**	-2.447e-03	3.256e-04	-7.515	6.17e-14*
**80+**	-2.857e-03	6.615e-04	-4.318	1.59e-05*
**EDUCATION**				
**SECONDARY**	1.459e-04	7.620e-04	0.191	0.84814
**TERTIARY SHORT**	-1.167e-03	1.041e-03	-1.121	0.26243
**TERTIARY LONG**	-2.087e-03	1.205e-03	-1.732	0.08332
**INCOME**				
**LOW**	-3.869e-05	6.680e-04	-0.058	0.95382
**MODERATE**	7.799e-04	7.477e-04	1.043	0.29696
**HIGH**	8.578e-04	8.696e-04	0.986	0.32397
**VERY HIGH**	2.576e-03	1.388e-03	1.856	0.06355
**BMI**				
**OVERWEIGHT**	1.447e-03	2.012e-04	7.193	6.79e-13*
**OBESE**	2.534e-03	2.409e-04	10.519	< 2e-16*
**OUTCOME: TOTAL FRESHWATER EUTROPHICATION ARISING FROM FOOD CONSUMPTION[B]**				
**SEX**	-1.127e-05	1.626e-05	-0.693	0.488317
**AGE**				
**50-59**	7.459e-06	4.628e-06	1.612	0.107088
**60-69**	-1.108e-06	4.911e-06	-0.226	0.821518
**70-79**	-1.838e-05	6.677e-06	-2.753	0.005910*
**80+**	-5.198e-05	1.353e-05	-3.843	0.000122*
**EDUCATION**				
**SECONDARY**	2.089e-05	1.568e-05	1.332	0.182975
**TERTIARY SHORT**	-8.941e-06	2.136e-05	-0.419	0.675483
**TERTIARY LONG**	-4.775e-05	2.489e-05	-1.918	0.055106
**INCOME**				
**LOW**	1.330e-05	1.371e-05	0.971	0.331804
**MODERATE**	3.253e-05	1.539e-05	2.114	0.034527*
**HIGH**	4.696e-05	1.793e-05	2.619	0.008834*
**VERY HIGH**	7.453e-05	2.848e-05	2.617	0.008878*
**BMI**				
**OVERWEIGHT**	3.499e-05	4.135e-06	8.463	< 2e-16*
**OBESE**	6.411e-05	4.950e-06	12.951	< 2e-16*
**OUTCOME: TOTAL GREENHOUSE GAS EMISSIONS ARISING FROM FOOD CONSUMPTION[C]**				
**SEX**	-1.102e-02	7.712e-02	-0.143	0.88635
**AGE**				
**50-59**	6.721e-02	2.199e-02	3.056	0.00225*
**60-69**	7.402e-02	2.337e-02	3.168	0.00154*
**70-79**	1.023e-02	3.176e-02	0.322	0.74725
**80+**	-1.091e-01	6.409e-02	-1.702	0.08886
**EDUCATION**				
**SECONDARY**	7.104e-02	7.432e-02	0.956	0.33921
**TERTIARY SHORT**	-7.688e-02	1.014e-01	-0.758	0.44848
**TERTIARY LONG**	-3.502e-01	1.174e-01	-2.983	0.00286*
**INCOME**				
**LOW**	5.984e-02	6.534e-02	0.916	0.35981
**MODERATE**	1.793e-01	7.323e-02	2.449	0.01434*
**HIGH**	2.110e-01	8.540e-02	2.471	0.01348*
**VERY HIGH**	3.447e-01	1.352e-01	2.549	0.01081*
**BMI**				
**OVERWEIGHT**	1.496e-01	1.966e-02	7.608	3.02e-14*
**OBESE**	2.696e-01	2.354e-02	11.453	< 2e-16*
**OUTCOME: TOTAL LAND USE ARISING FROM FOOD CONSUMPTION[D]**				
**SEX**	-3.806e-03	8.041e-02	-0.047	0.962249
**AGE**				

**50-59**	-8.485e-02	2.308e-02	-3.676	0.000238*
**60-69**	-1.618e-01	2.446e-02	-6.615	3.90e-11*
**70-79**	-2.476e-01	3.323e-02	-7.450	1.01e-13*
**80+**	-3.763e-01	6.712e-02	-5.605	2.13e-08*
**EDUCATION**				
**SECONDARY**	-6.666e-02	7.786e-02	-0.856	0.391925
**TERTIARY SHORT**	-1.389e-01	1.063e-01	-1.307	0.191162
**TERTIARY LONG**	-3.219e-01	1.230e-01	-2.617	0.008890*
**INCOME**				
**LOW**	1.966e-02	6.813e-02	0.289	0.772914
**MODERATE**	8.675e-02	7.619e-02	1.139	0.254852
**HIGH**	1.245e-01	8.896e-02	1.399	0.161748
**VERY HIGH**	2.986e-01	1.432e-01	2.086	0.037035*
**BMI**				
**OVERWEIGHT**	1.742e-01	2.058e-02	8.465	< 2e-16*
**OBESE**	3.580e-01	2.464e-02	14.527	< 2e-16*
**OUTCOME: TOTAL WATER USE ARISING FROM FOOD CONSUMPTION[E]**				
**SEX**	-2.419e-02	1.382e-02	-1.750	0.080149
**AGE**				
**50-59**	-1.185e-02	3.936e-03	-3.012	0.002602*
**60-69**	-1.452e-02	4.180e-03	-3.474	0.000516*
**70-79**	-1.631e-02	5.699e-03	-2.861	0.004228 *
**80+**	5.613e-03	1.155e-02	0.486	0.627051
**EDUCATION**				
**SECONDARY**	2.518e-02	1.333e-02	1.890	0.058847
**TERTIARY SHORT**	1.572e-02	1.833e-02	0.858	0.391101
**TERTIARY LONG**	2.445e-02	2.127e-02	1.150	0.250372
**INCOME**				
**LOW**	-3.199e-03	1.169e-02	-0.274	0.784289
**MODERATE**	1.367e-02	1.313e-02	1.041	0.297998
**HIGH**	-1.975e-04	1.528e-02	-0.013	0.989688
**VERY HIGH**	-5.077e-03	2.456e-02	-0.207	0.836251
**BMI**				
**OVERWEIGHT**	-5.259e-04	3.523e-03	-0.149	0.881322
**OBESE**	-1.616e-03	4.207e-03	-0.384	0.700873

***P<0.05 MODEL INCLUDED INTERACTION EFFECT BETWEEN SEX:EDUCATION:INCOME AND ADJUSTED FOR SMOKING STATUS, PHYSICAL ACTIVITY STATUS, AND ENERGY INTAKE. REFERENCE CATEGORIES WERE FEMALE, AGE 40-49, PRIMARY EDUCATION, VERY LOW INCOME, AND NORMAL WEIGHT.**
A 68.2% OF THE VARIABILITY IN TOTAL IMPACT CAN BE EXPLAINED BY THE MODEL (F=441.5, P<0.05).
B 70.9% OF THE VARIABILITY IN TOTAL IMPACT CAN BE EXPLAINED BY THE MODEL (F=499.8, P<0.05).
C 68.3% OF THE VARIABILITY IN TOTAL IMPACT CAN BE EXPLAINED BY THE MODEL (F=439.5, P<0.05).
D 67.0% OF THE VARIABILITY IN TOTAL IMPACT CAN BE EXPLAINED BY THE MODEL (F=415.4, P<0.05).
E 53.4% OF THE VARIABILITY IN TOTAL IMPACT CAN BE EXPLAINED BY THE MODEL (F=237.0, P<0.05).

S6. Pairwise comparisons for each interaction category of interest

Greenhouse Gas Emissions

	Male*Primary Education*Very High Income	Male*Tertiary Long Education*Very Low Income	Male*Tertiary Long Education*Very High Income	Female*Primary Education*Very Low Income	Female*Primary Education*Very High Income	Female*Tertiary Long Education*Very Low Income	Female*Tertiary Long Education*Very High Income
Male*Primary Education*Very Low Income	+	+	+	+	+	-	+
Male*Primary Education*Very High Income		-	-	-	-	-	-
Male*Tertiary Long Education*Very Low Income			+	-	+	-	+
Male*Tertiary Long Education*Very High Income				-	+	-	+
Female*Primary Education*Very Low Income					+	-	+
Female*Primary Education*Very High Income						-	-
Female*Tertiary Long Education*Very Low Income							+

Terrestrial Acidification

	Male*Primary Education*Very High Income	Male*Tertiary Long Education*Very Low Income	Male*Tertiary Long Education*Very High Income	Female*Primary Education*Very Low Income	Female*Primary Education*Very High Income	Female*Tertiary Long Education*Very Low Income	Female*Tertiary Long Education*Very High Income
Male*Primary Education*Very Low Income	+	+	+	+	+	-	+
Male*Primary Education*Very High Income		-	-	-	-	-	-
Male*Tertiary Long Education*Very Low Income			-	-	+	-	-
Male*Tertiary Long Education*Very High Income				-	+	-	+
Female*Primary Education*Very Low Income					+	-	+
Female*Primary Education*Very High Income						-	-
Female*Tertiary Long Education*Very Low Income							+

Freshwater Eutrophication

	Male*Primary Education*Very High Income	Male*Tertiary Long Education*Very Low Income	Male*Tertiary Long Education*Very High Income	Female*Primary Education*Very Low Income	Female*Primary Education*Very High Income	Female*Tertiary Long Education*Very Low Income	Female*Tertiary Long Education*Very High Income
Male*Primary Education*Very Low Income	+	+	+	+	+	-	+
Male*Primary Education*Very High Income		-	-	-	-	-	-
Male*Tertiary Long Education*Very Low Income			-	-	+	-	+
Male*Tertiary Long Education*Very High Income				-	+	-	+
Female*Primary Education*Very Low Income					+	-	+
Female*Primary Education*Very High Income						-	-
Female*Tertiary Long Education*Very Low Income							+

Land Use

	Male*Primary Education*Very High Income	Male*Tertiary Long Education*Very Low Income	Male*Tertiary Long Education*Very High Income	Female*Primary Education*Very Low Income	Female*Primary Education*Very High Income	Female*Tertiary Long Education*Very Low Income	Female*Tertiary Long Education*Very High Income
Male*Primary Education*Very Low Income	+	-	+	+	+	-	+
Male*Primary Education*Very High Income		-	-	-	-	-	-
Male*Tertiary Long Education*Very Low Income			+	+	+	-	+
Male*Tertiary Long Education*Very High Income				-	+	-	-
Female*Primary Education*Very Low Income					+	-	+
Female*Primary Education*Very High Income						-	-
Female*Tertiary Long Education*Very Low Income							+

Water Use

	Male*Primary Education*Very High Income	Male*Tertiary Long Education*Very Low Income	Male*Tertiary Long Education*Very High Income	Female*Primary Education*Very Low Income	Female*Primary Education*Very High Income	Female*Tertiary Long Education*Very Low Income	Female*Tertiary Long Education*Very High Income
Male*Primary Education*Very Low Income	+	+	+	+	+	+	+
Male*Primary Education*Very High Income		+	+	+	+	+	+
Male*Tertiary Long Education*Very Low Income			-	-	-	+	+
Male*Tertiary Long Education*Very High Income				+	-	+	-
Female*Primary Education*Very Low Income					-	+	+
Female*Primary Education*Very High Income						+	+
Female*Tertiary Long Education*Very Low Income							+

The figure is organized from row to column. Lighter colors indicate weaker associations, while darker colors represent stronger associations. Positive associations are marked with a "+" sign, and negative associations with a "-" sign. Predicted differences between interacting groups are calculated by using pairwise function with a Bonferroni correction. Red cells indicate statistical significance with $p < 0.05$, while blue cells indicate $p \geq 0.05$. The color scale is derived using quantile-based partitioning.

S7. INTERACTION EFFECTS IN REGRESSION MODEL

	Effect Estimate	Standard Error	T Statistics
**OUTCOME: TOTAL GREENHOUSE GAS EMISSIONS ARISING FROM FOOD CONSUMPTION**			
**Male*Secondary Education**	-1.971e-01	1.270e-01	-1.553
**Male*Tertiary Education Short**	4.414e-02	1.806e-01	0.244
**Male*Tertiary Education Long**	4.648e-01	2.180e-01	2.132**
**Male*Low Income**	-2.337e-02	1.033e-01	-0.226
**Male*Moderate Income**	-1.370e-01	1.115e-01	-1.229
**Male*High Income**	5.455e-02	1.223e-01	0.446
**Male*Very High Income**	1.428e-01	1.831e-01	0.780
**Secondary Education*Low Income**	-2.696e-02	9.732e-02	-0.277
**Tertiary Education Short*Low Income**	1.705e-01	1.244e-01	1.370
**Tertiary Education Long*Low Income**	3.911e-01	1.336e-01	2.927**
**Secondary Education*Moderate Income**	-7.102e-02	1.050e-01	-0.677
**Tertiary Education Short*Moderate Income**	6.201e-02	1.345e-01	0.461
**Tertiary Education Long*Moderate Income**	2.652e-01	1.388e-01	1.911
**Secondary Education*High Income**	-7.419e-02	1.122e-01	-0.662
**Tertiary Education Short*High Income**	2.149e-02	1.338e-01	0.161
**Tertiary Education Long*High Income**	3.660e-01	1.428e-01	2.563**
**Secondary Education*Very High Income**	-1.182e-01	1.631e-01	-0.725
**Tertiary Education Short*Very High Income**	2.632e-03	1.696e-01	0.016
**Tertiary Education Long*Very High Income**	3.229e-01	1.750e-01	1.845
**Male*Secondary Education*Low Income**	8.950e-02	1.579e-01	0.567
**Male*Tertiary Education Short*Low Income**	-5.922e-02	2.120e-01	-0.279
**Male*Tertiary Education Long*Low Income**	-6.597e-01	2.453e-01	-2.689**
**Male*Secondary Education*Moderate Income**	3.344e-01	1.657e-01	2.018**
**Male*Tertiary Education Short*Moderate Income**	3.357e-02	2.172e-01	0.155
**Male*Tertiary Education Long*Moderate Income**	-4.108e-01	2.443e-01	-1.682
**Male*Secondary Education*High Income**	1.308e-01	1.696e-01	0.771
**Male*Tertiary Education Short*High Income**	-6.094e-02	2.154e-01	-0.283
**Male*Tertiary Education Long*High Income**	-6.206e-01	2.456e-01	-2.527**
**Male*Secondary Education*Very High Income**	9.832e-02	2.280e-01	0.431
**Male*Tertiary Education Short*Very High Income**	-1.234e-01	2.541e-01	-0.486
**OUTCOME: TOTAL TERRESTRIAL ACIDIFICATION ARISING FROM FOOD CONSUMPTION**			
**Male*Secondary Education**	2.251e-04	1.296e-03	0.174
**Male*Tertiary Education Short**	2.819e-03	1.855e-03	1.520
**Male*Tertiary Education Long**	5.295e-03	2.270e-03	2.332**
**Male*Low Income**	1.591e-03	1.057e-03	1.505
**Male*Moderate Income**	4.033e-04	1.140e-03	0.354
**Male*High Income**	1.867e-03	1.249e-03	1.495
**Male*Very High Income**	1.865e-03	1.875e-03	0.994
**Secondary Education*Low Income**	4.218e-04	9.961e-04	0.423
**Tertiary Education Short*Low Income**	2.149e-03	1.277e-03	1.683
**Tertiary Education Long*Low Income**	2.438e-03	1.371e-03	1.778
**Secondary Education*Moderate Income**	-3.376e-04	1.074e-03	-0.314
**Tertiary Education Short*Moderate Income**	1.336e-03	1.378e-03	0.969
**Tertiary Education Long*Moderate Income**	1.409e-03	1.424e-03	0.989

**Secondary Education*High Income**	-3.492e-05	1.146e-03	-0.030
**Tertiary Education Short*High Income**	1.136e-03	1.370e-03	0.830
**Tertiary Education Long*High Income**	2.398e-03	1.463e-03	1.639
**Secondary Education*Very High Income**	-9.777e-04	1.675e-03	-0.584
**Tertiary Education Short*Very High Income**	-2.902e-04	1.742e-03	-0.167
**Tertiary Education Long*Very High Income**	1.419e-03	1.798e-03	0.789
**Male*Secondary Education*Low Income**	-1.670e-03	1.611e-03	-1.037
**Male*Tertiary Education Short*Low Income**	-3.547e-03	2.176e-03	-1.630
**Male*Tertiary Education Long*Low Income**	-7.437e-03	2.545e-03	-2.922**
**Male*Secondary Education*Moderate Income**	1.118e-03	1.689e-03	0.662
**Male*Tertiary Education Short*Moderate Income**	-2.711e-03	2.227e-03	-1.217
**Male*Tertiary Education Long*Moderate Income**	-5.136e-03	2.534e-03	-2.027**
**Male*Secondary Education*High Income**	-7.581e-04	1.730e-03	-0.438
**Male*Tertiary Education Short*High Income**	-3.546e-03	2.208e-03	-1.606
**Male*Tertiary Education Long*High Income**	-7.537e-03	2.548e-03	-2.958**
**Male*Secondary Education*Very High Income**	-4.094e-04	2.335e-03	-0.175
**Male*Tertiary Education Short*Very High Income**	-2.943e-03	2.607e-03	-1.129
**OUTCOME: TOTAL FRESHWATER EUTROPHICATION ARISING FROM FOOD CONSUMPTION**			
**Male*Secondary Education**	-2.393e-05	2.667e-05	-0.897
**Male*Tertiary Education Short**	2.886e-05	3.805e-05	0.759
**Male*Tertiary Education Long**	9.244e-05	4.665e-05	1.981**
**Male*Low Income**	-1.551e-07	2.173e-05	-0.007
**Male*Moderate Income**	-1.019e-05	2.344e-05	-0.435
**Male*High Income**	7.885e-06	2.569e-05	0.307
**Male*Very High Income**	1.214e-05	3.857e-05	0.315
**Secondary Education*Low Income**	-3.296e-06	2.049e-05	-0.161
**Tertiary Education Short*Low Income**	4.160e-05	2.621e-05	1.587
**Tertiary Education Long*Low Income**	6.357e-05	2.828e-05	2.248**
**Secondary Education*Moderate Income**	-1.321e-05	2.210e-05	-0.598
**Tertiary Education Short*Moderate Income**	1.631e-05	2.824e-05	0.578
**Tertiary Education Long*Moderate Income**	3.357e-05	2.938e-05	1.143
**Secondary Education*High Income**	-2.806e-05	2.362e-05	-1.188
**Tertiary Education Short*High Income**	-7.766e-06	2.817e-05	-0.276
**Tertiary Education Long*High Income**	5.038e-05	3.020e-05	1.669
**Secondary Education*Very High Income**	-3.649e-05	3.440e-05	-1.061
**Tertiary Education Short*Very High Income**	-9.013e-06	3.573e-05	-0.252
**Tertiary Education Long*Very High Income**	4.012e-05	3.698e-05	1.085
**Male*Secondary Education*Low Income**	1.957e-06	3.315e-05	0.059
**Male*Tertiary Education Short*Low Income**	-3.478e-05	4.465e-05	-0.779
**Male*Tertiary Education Long*Low Income**	-1.190e-04	5.225e-05	-2.278**
**Male*Secondary Education*Moderate Income**	4.801e-05	3.476e-05	1.381
**Male*Tertiary Education Short*Moderate Income**	-4.074e-05	4.567e-05	-0.892
**Male*Tertiary Education Long*Moderate Income**	-8.536e-05	5.206e-05	-1.640
**Male*Secondary Education*High Income**	2.347e-05	3.561e-05	0.659
**Male*Tertiary Education Short*High Income**	-1.883e-05	4.533e-05	-0.416
**Male*Tertiary Education Long*High Income**	-1.346e-04	5.236e-05	-2.571**
**Male*Secondary Education*Very High Income**	2.188e-05	4.803e-05	0.456

**Male*Tertiary Education Short*Very High Income**	-3.392e-05	5.355e-05	-0.633
**OUTCOME: TOTAL LAND USE ARISING FROM FOOD CONSUMPTION**			
**Male*Secondary Education**	-2.254e-02	1.325e-01	-0.170
**Male*Tertiary Education Short**	1.329e-01	1.922e-01	0.692
**Male*Tertiary Education Long**	2.524e-01	2.317e-01	1.089
**Male*Low Income**	8.981e-02	1.079e-01	0.832
**Male*Moderate Income**	1.971e-02	1.163e-01	0.170
**Male*High Income**	1.435e-01	1.276e-01	1.124
**Male*Very High Income**	7.573e-02	1.935e-01	0.391
**Secondary Education*Low Income**	6.562e-02	1.017e-01	0.645
**Tertiary Education Short*Low Income**	8.263e-02	1.305e-01	0.633
**Tertiary Education Long*Low Income**	2.052e-01	1.400e-01	1.465
**Secondary Education*Moderate Income**	7.172e-02	1.097e-01	0.654
**Tertiary Education Short*Moderate Income**	1.149e-01	1.405e-01	0.818
**Tertiary Education Long*Moderate Income**	1.006e-01	1.453e-01	0.692
**Secondary Education*High Income**	-1.582e-03	1.172e-01	-0.013
**Tertiary Education Short*High Income**	4.570e-02	1.399e-01	0.327
**Tertiary Education Long*High Income**	1.641e-01	1.495e-01	1.098
**Secondary Education*Very High Income**	-9.735e-02	1.722e-01	-0.565
**Tertiary Education Short*Very High Income**	-1.215e-01	1.790e-01	-0.679
**Tertiary Education Long*Very High Income**	5.207e-02	1.846e-01	0.282
**Male*Secondary Education*Low Income**	-1.509e-02	1.649e-01	-0.092
**Male*Tertiary Education Short*Low Income**	-4.195e-02	2.248e-01	-0.187
**Male*Tertiary Education Long*Low Income**	-3.956e-01	2.601e-01	-1.521
**Male*Secondary Education*Moderate Income**	1.899e-01	1.729e-01	1.099
**Male*Tertiary Education Short*Moderate Income**	-9.449e-02	2.298e-01	-0.411
**Male*Tertiary Education Long*Moderate Income**	-2.087e-01	2.588e-01	-0.806
**Male*Secondary Education*High Income**	6.539e-02	1.771e-01	0.369
**Male*Tertiary Education Short*High Income**	-1.329e-01	2.280e-01	-0.583
**Male*Tertiary Education Long*High Income**	-3.689e-01	2.602e-01	-1.418
**Male*Secondary Education*Very High Income**	1.404e-01	2.401e-01	0.585
**Male*Tertiary Education Short*Very High Income**	-4.007e-02	2.698e-01	-0.149
**OUTCOME: TOTAL WATER USE ARISING FROM FOOD CONSUMPTION**			
**Male*Secondary Education**	-2.608e-02	2.270e-02	-1.149
**Male*Tertiary Education Short**	-2.370e-02	3.301e-02	-0.718
**Male*Tertiary Education Long**	1.152e-02	3.982e-02	0.289
**Male*Low Income**	-6.885e-03	1.841e-02	-0.374
**Male*Moderate Income**	-3.343e-02	1.987e-02	-1.683
**Male*High Income**	-1.140e-02	2.180e-02	-0.523
**Male*Very High Income**	5.596e-03	3.275e-02	0.171
**Secondary Education*Low Income**	1.603e-03	1.742e-02	0.092
**Tertiary Education Short*Low Income**	1.436e-02	2.245e-02	0.640
**Tertiary Education Long*Low Income**	2.586e-02	2.417e-02	1.070
**Secondary Education*Moderate Income**	-3.219e-02	1.882e-02	-1.711
**Tertiary Education Short*Moderate Income**	-2.522e-03	2.419e-02	-0.104
**Tertiary Education Long*Moderate Income**	1.049e-02	2.508e-02	0.418
**Secondary Education*High Income**	-1.590e-02	2.008e-02	-0.792

**Tertiary Education Short*High Income**	-1.186e-02	2.411e-02	-0.492
**Tertiary Education Long*High Income**	2.138e-02	2.578e-02	0.829
**Secondary Education*Very High Income**	-7.981e-03	2.951e-02	-0.270
**Tertiary Education Short*Very High Income**	1.323e-02	3.076e-02	0.430
**Tertiary Education Long*Very High Income**	3.268e-02	3.177e-02	1.029
**Male*Secondary Education*Low Income**	-2.804e-03	2.815e-02	-0.100
**Male*Tertiary Education Short*Low Income**	2.394e-02	3.850e-02	0.622
**Male*Tertiary Education Long*Low Income**	-3.929e-02	4.457e-02	-0.882
**Male*Secondary Education*Moderate Income**	4.294e-02	2.952e-02	1.455
**Male*Tertiary Education Short*Moderate Income**	3.758e-02	3.938e-02	0.954
**Male*Tertiary Education Long*Moderate Income**	-1.149e-03	4.442e-02	-0.026
**Male*Secondary Education*High Income**	2.169e-02	3.023e-02	0.718
**Male*Tertiary Education Short*High Income**	3.372e-02	3.908e-02	0.863
**Male*Tertiary Education Long*High Income**	-3.188e-02	4.466e-02	-0.714
**Male*Secondary Education*Very High Income**	-2.503e-03	4.077e-02	-0.061
**Male*Tertiary Education Short*Very High Income**	2.657e-03	4.596e-02	0.058

*Model included age, sex, education, income, body mass index, interaction effects between sex:education:income, and adjusted for smoking status, physical activity, and energy intake. Reference categories were female, primary education, and very low income. Education is categorized into four levels; primary (up to 10 years of schooling), secondary education (a minimum of 3 years), tertiary short (college/university less than 4 years), and tertiary long (college/university 4 years or more). Income is given in Norwegian kroners, and categorized into five levels; <=350000 (very low), 351000 – 550000 (low), 551000 – 750000 (moderate), 750000 – 1000000 (high), 1000000 + (very high). **statistically significant (p < 0.05).*

S8. Total number of individuals in each intersectional group

	Greenhouse gas emissions	Terrestrial Acidification	Freshwater Eutrophication	Land Use	Water Use
**Male*Primary Education*Very Low Income**	207	207	206	209	209
**Male*Primary Education*Very High Income**	63	64	63	62	67
**Male*Tertiary Long Education*Very Low Income**	25	24	24	24	24
**Male*Tertiary Long Education*Very High Income**	716	720	719	717	713
**Female*Primary Education*Very Low Income**	380	384	381	386	381
**Female*Primary Education*Very High Income**	47	47	47	46	46
**Female*Tertiary Long Education*Very Low Income**	67	67	66	67	66
**Female*Tertiary Long Education*Very High Income**	849	850	846	849	845
